# Effect of Storage Time on the Structure and Performance of Hybrid Polysulfone Membranes with Graphene Nanoplatelets

**DOI:** 10.1021/acsomega.5c00467

**Published:** 2025-05-28

**Authors:** Nathália Ferronato Livinalli, Jocelei Duarte, Wendel Paulo Silvestre, Camila Baldasso

**Affiliations:** Postgraduate Program in Process Engineering and Technologies (PGEPROTEC), 58802 University of Caxias do Sul, Street Francisco Getúlio Vargas. 1130, Petrópolis, Caxias do Sul, RS 95070-560, Brazil

## Abstract

This study investigated the impact of time on the structure and performance of hybrid polysulfone (PSU) membranes containing graphene nanoplatelets (GNP), synthesized in 2022. FTIR, SEM, TGA, and BET were used to analyze structural and functional changes after two years of storage. The membranes exhibited distinct behaviors depending on GNP concentration and usage conditions. Pristine membranes showed larger average pore size (16–17 nm) and lower surface area (4.7–16.9 m^2^·g^–1^), whereas compacted membranes exhibited smaller pores (3.6–5.4 nm) and higher surface area (up to 322.7 m^2^·g^–1^). Compaction and storage drastically reduced hydraulic permeability, attributed to fouling, nanoparticle agglomeration, and oxidative degradation of the polymeric matrix. Furthermore, pristine membranes were tested after two years and ruptured during compaction and permeability tests, revealing mechanical fragility caused by aging. FTIR results suggested chemical degradation, with changes in absorption band intensities. SEM micrographs displayed stress marks, cracks, and biological contamination on used membranes, while pristine membranes showed less resilient morphology. TGA analyses revealed a reduction in maximum degradation temperature, indicating thermal stability loss. BET results highlighted the impact of usage and time on pore redistribution and surface area. This research emphasizes the need to optimize storage conditions and explore strategies to improve membranes’ thermal and mechanical stability. Future studies should investigate chemical modifications to the polymeric matrix and evaluate new additives to mitigate degradation during long-term storage.

## Graphical abstract

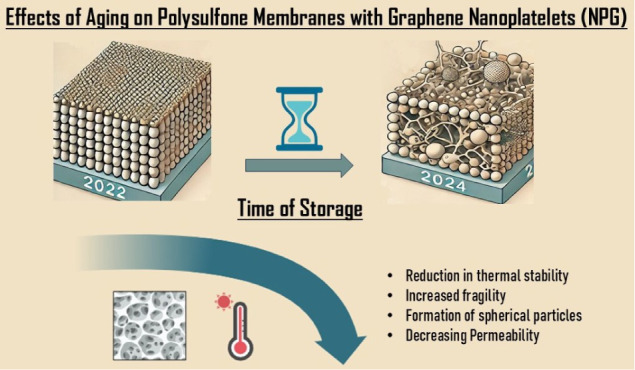

## 1 Introduction

The application of membrane separation systems has significantly grown across various industrial sectors, including wastewater treatment, water purification, gas separation, and concentration and distillation processes in chemical and pharmaceutical industries. These systems are preferred for their high efficiency, low energy consumption, and operational simplicity. However, the membranes used in such processes are susceptible to degradation over time, which can compromise their performance and durability.^1–3^ Factors such as chemical degradation, oxidation, fouling (pore-clogging), concentration polarization (substance accumulation on the surface), thermal aging, and mechanical distortions directly affect membrane morphology and efficiency. Polymeric degradation, in particular, is critical, as it reduces the mobility of polymer chains, one of the leading results of aging, compromising the mechanical properties and stability of membranes. Chain mobility reduction can result from cross-linking or bond breaking during oxidation, leading to stiffened structures and decreased material flexibility. This may result in a loss of the membranes’ ability to adapt to pressure and temperature variations, directly impacting essential properties such as permeability and flux.^4,5^

Reduced polymer chain mobility decreases efficiency in molecule passage through the membrane, reducing permeability. Additionally, restricted chain movement hampers the reversal of deformations caused by pressure or temperature, increasing flow resistance and compromising separation system performance. These changes are often observed in polymer membranes such as Nafion and polysulfone (PSU), where oxidation and thermal aging alter structure and ion or molecule transport properties.^6–9^

The physical aging of polymeric membranes is a widely studied phenomenon that significantly impacts their performance, especially in gas separation applications. Zhang and Wang investigated the physical aging in polysulfone membranes and observed a reduction in the diffusion coefficient over time, indicating a decrease in free volume within the polymer matrix.^10^ This directly affects the mobility of polymer chains, with dye diffusion following a power-law relationship over time, suggesting that the chains progressively become more rigid. The effect was more pronounced near the glass transition temperature (*T*_g_) and disappeared below 165 °C. Rowe et al. explored aging in thin polysulfone films used for gas separation and found a decrease in permeability and an increase in selectivity over time. Aging was accelerated in films exposed to CO_2_, suggesting that initial swelling followed by structural relaxation contributes to membrane densification.^11^

Regarding mitigation, Bakhtin et al. reviewed strategies to delay physical aging, emphasizing the importance of cross-linking and the use of nanoparticles.^12^ Membranes with a high free volume fraction, such as PIMs, age more rapidly, but techniques like nanoparticle incorporation can help maintain permeability over time. Furthermore, ultrathin membranes (<1 μm) exhibit greater chain mobility and age more quickly. Postmodification approaches, including cross-linking of PIMs, have also successfully mitigated physical aging while maintaining gas separation performance. Physical aging results in a continuous decrease in permeability, especially for larger penetrants, and densification over time affects long-term performance. Additionally, the excess free volume in the glassy state of a polymer can influence its sorption capacity and thermal history-dependent behavior, which are key features of physical aging.^13–15^

Livinalli et al. synthesized PSU membranes with GNP via phase inversion, showing excellent retention rates. However, permeability tests revealed a time effect, with reduced water permeate flux between 2022 and 2023. This suggests that structural alterations over time, possibly caused by reduced polymer chain mobility, impact efficiency, and functionality.^16^

This work aims to analyze how degradation factors, such as decreased polymer chain mobility, affect membrane properties over time. Tests were conducted on membranes synthesized after two years of storage using techniques like scanning electron microscopy (SEM), BET analysis, thermogravimetric analysis (TGA), FTIR, and permeability tests. The objective was to investigate how storage time affected membrane structure, porosity, permeability, thermal resistance, and chemical bonds.

## 2 Materials and Methods

### 2.1 Materials

The materials used to study time effects on membranes were the membranes synthesized by Livinalli et al., with properties described in Table 1.^16^ The membranes were stored in plastic bags inside drawers in the laboratory, under 23 ± 5% relative humidity and at a temperature of 23 ± 2 °C. These included PSU membranes with 0.5, 1.0, and 1.5 wt % GNP. Distilled water was used for permeability tests.

**Table 1:** Properties of Membranes Synthesized in 2022

Property	Zero	0.5 wt % GNP	1.0 wt % GNP	1.5 wt % GNP
Permeability – *L*_p_ (L·m^–2^·h^–1^·bar^–1^)	6.78 ± 1.82	2.44 ± 0.65	0.32 ± 0.04	0.10 ± 0.04
Average pore radius (Å)	41.7	38.1	57.8	35.3

### 2.2 Methods

The methods selected for comparing membrane characterization between 2022 and 2024 included Fourier Transform Infrared Spectroscopy (FTIR) to identify degradation points and the formation of new absorption bands. The technique employed was attenuated total reflection (ATR), within the wavenumber range of 650–4000 cm^–1^, conducting 32 scans with a resolution of 4.0 cm^–1^, using a PerkinElmer Spectrum 400 instrument.

The morphology was compared through transverse and surface images obtained using a field emission scanning electron microscope (FESEM) from Tescan (Czech Republic), model MIRA-3, operating at an acceleration voltage of 10 kV. Magnifications ranged from 2,000× to 20,000× for transverse images and from 5,000× to 100,000× for surface images. For cross-sectional observation, the membranes were fractured after exposure to liquid nitrogen (−196 °C). The samples were sputter-coated with a thin gold layer for 2 min before characterization.

Mass loss as a function of temperature and the maximum degradation point was evaluated through thermogravimetric analysis (TGA) using a Netzsch TG 209 Tarsus F3 instrument. In the TGA and FTIR analyses, the equipment used in 2022 was replaced due to the unavailability of the same technologies, but the analytical parameters were maintained. BET (Brunauer–Emmett–Teller) analysis was performed to determine membrane porosity using the NOVA 1200e Surface Area and Pore Size Analyzer from Quantachrome Instruments to complement the studies. The samples were degassed at 150 °C for 20 h under a nitrogen atmosphere.

The hydraulic permeability test system was maintained, and the experimental setup is shown in Figure 1, illustrating the application of the membrane module. The setup included a 2 L feed tank containing distilled water connected to a flat-sheet stainless steel membrane module. The feed solution temperature was kept constant at 25 ± 2 °C in all experiments, monitored by a thermometer installed in the feed tank. During permeability tests, the permeate and concentrate streams were recirculated. The transmembrane pressure was controlled using a valve located at the concentrate outlet and monitored via two pressure gauges positioned at the inlet and outlet of the concentrate. Permeate flow measurements were performed using a graduated cylinder, with samples collected for 1 min.

**Figure 1:**
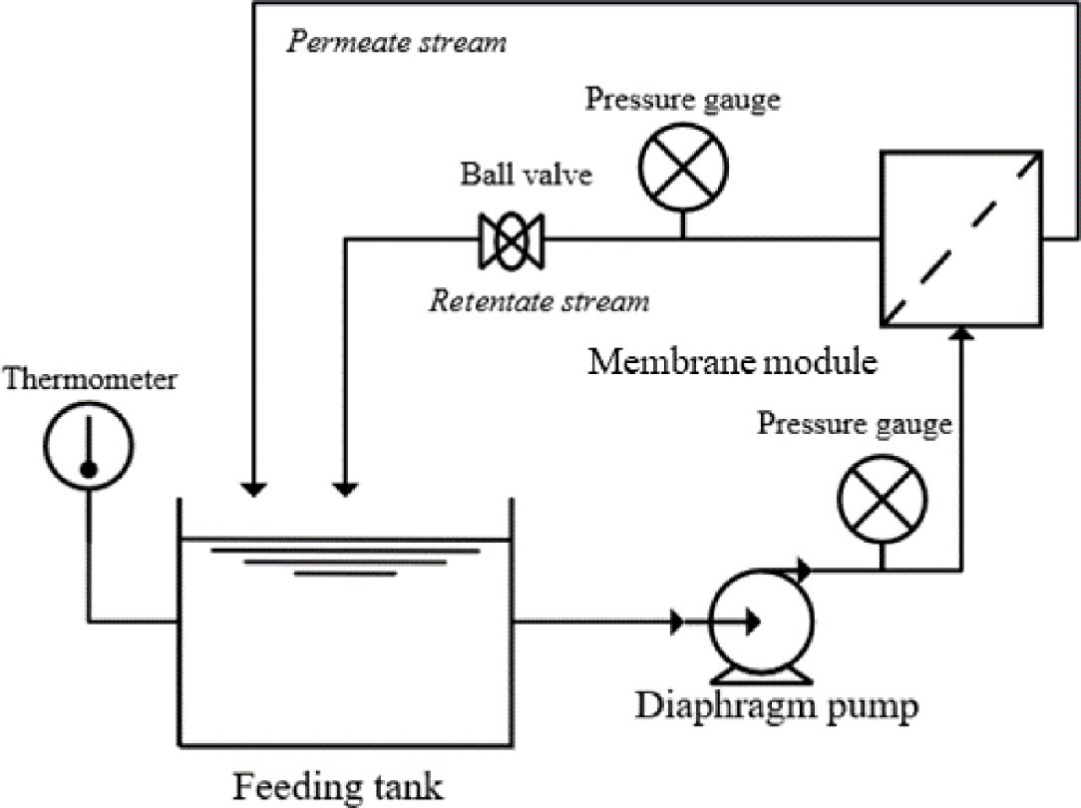
Flowchart of the system used in membrane permeability tests. Source: Livinalli et al.^16^

The permeability test aimed to determine the hydraulic permeability of the membrane based on the permeate flux obtained and the applied transmembrane pressure. The permeate flux (*J*_p_, L·m^–2^·h^–1^) and hydraulic permeability (*L*_p_, L·m^–2^·h^–1^·bar^–1^) were calculated following the methodology described by Mulder.^17^

The characterization analyses were applied to eight samples. The previously used and compacted samples in 2022 were labeled as samples A1 to A4, and the samples tested for the first time were labeled as A5 to A8. The results were compared with those obtained in 2022 with the same membranes, as discussed by Livinalli et al.^16^

## 3 Results and Discussion

Through the mentioned methodology, the following results aim to present and discuss the effect of time on the synthesized PSU membranes with NPG. The effect can be identified by the degradation of polysulfone or external contamination effects. The membranes were stored dry in plastic bags inside a drawer, without exposure to UV radiation, under 23% relative humidity and at a temperature of 23 °C, for two years.

Polysulfone degradation can occur through oxidative means when in contact with oxidizing agents, hydrolysis when in contact with acidic and basic solutions, thermal, mechanical, and photodegradation. The compacted membranes may have been affected by mechanical degradation, which reduces the structural integrity of the membrane.^18^

### 3.1 Comparison of FTIR

FTIR analyses were performed to compare the spectra obtained under different conditions. Figure 2 presents (A) absorption bands of the membranes analyzed in 2022 – samples A1 to A4, (B) absorption bands of the membranes used in 2024 – samples A1 to A4, and (C) absorption bands of the unused membranes in 2024 – samples A5 to A8.

**Figure 2:**
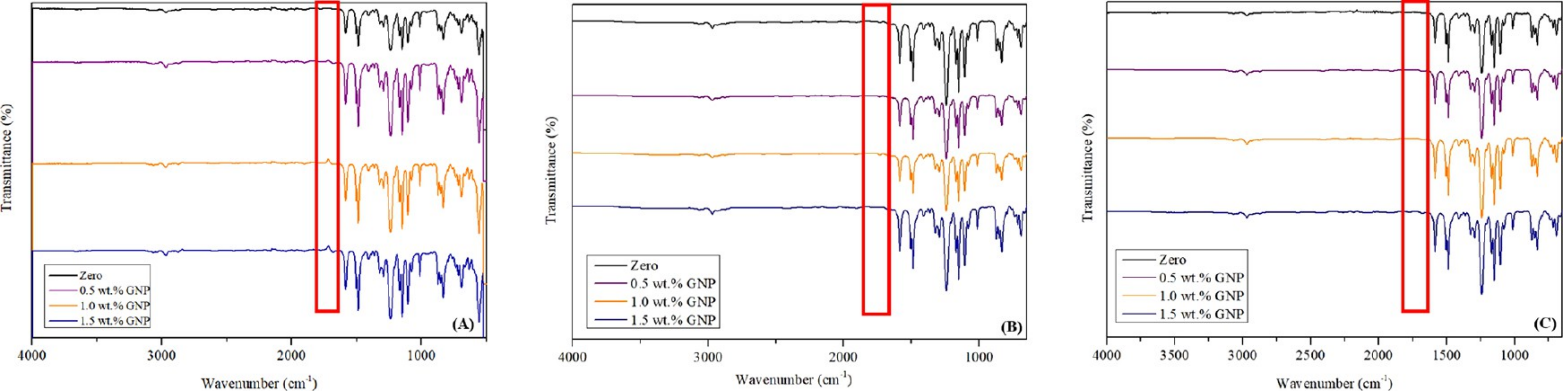
FTIR spectra of the membranes: (A) absorption bands of the membranes analyzed in 2022 – samples A to D, (B) absorption bands of the membranes used in 2024 – samples A1 to A4, and (C) absorption bands of the unused membranes in 2024 – samples A5 to A8.^16^

The main wavenumbers identified as characteristic of polysulfone (PSU) are 2976 cm^–1^ (C–H axial stretching), 690 cm^–1^, 1322–1292 cm^–1^, 1592–1583 cm^–1^ (C–S, C–O, and C=C aromatic stretching), 1078 cm^–1^ (S=O), and 1486–1363 cm^–1^ (C–H and CH_3_ bending). The band at 1684 cm^–1^, associated with the axial vibration of the C=O aromatic bond, is attributed to the presence of NPG. The intensity of the bands is a critical characteristic for evaluating degradation processes or chemical modifications, highlighted in red. Although no new bonds were identified in spectra B and C, an increase in band intensity was observed when compared to spectrum A.^19–21^

Specifically, the bands associated with C–S, C–O, and C=C aromatic bonds showed higher transmittance in spectra B and C than spectrum A, indicating potential molecular density or organization changes. Additionally, the band at 1684 cm^–1^ (C=O of NPG) disappeared when comparing spectrum A with spectra B and C. This change may be related to degradation processes or chemical interactions during storage. It is important to highlight that the retention, compaction, and permeability tests performed in 2022 did not alter the spectral characteristics of the membranes, as spectra B and C remained similar, indicating the structural stability of the membranes used under controlled conditions.

The increase in absorption intensity demonstrates lower light absorption at the specific wavenumber, which may indicate degradation or aging of the sample. Over time, chemical bonds may weaken, resulting in lower radiation absorption in the corresponding range.^22^ Chokki et al. studied the degradation of ultrafiltration membranes made of polyethersulfone (PES) and polyvinylpyrrolidone (PVP) by exposing the membranes to hypochlorite solutions. Chemical degradation primarily occurred due to the rupture of S–O and C–S bonds, and more rigid groups were found in the polymeric structure, which may reduce chain mobility.^23^

### 3.2 Comparison of MEV

SEM analyses compared the results obtained in 2022 with those from 2024. Figures 3, 4, and 5 show the micrographs of the membrane surfaces. Samples A, B, C, and D correspond to pieces of the membranes analyzed in 2022. Samples A1, A2, A3, and A4 are pieces of the membranes used in the compaction tests, analyzed in 2024. Samples A5, A6, A7, and A8 represent pieces of membranes analyzed in 2024 but never used. All micrographs were obtained from different regions of the membranes selected based on specific points of interest.

**Figure 3:**
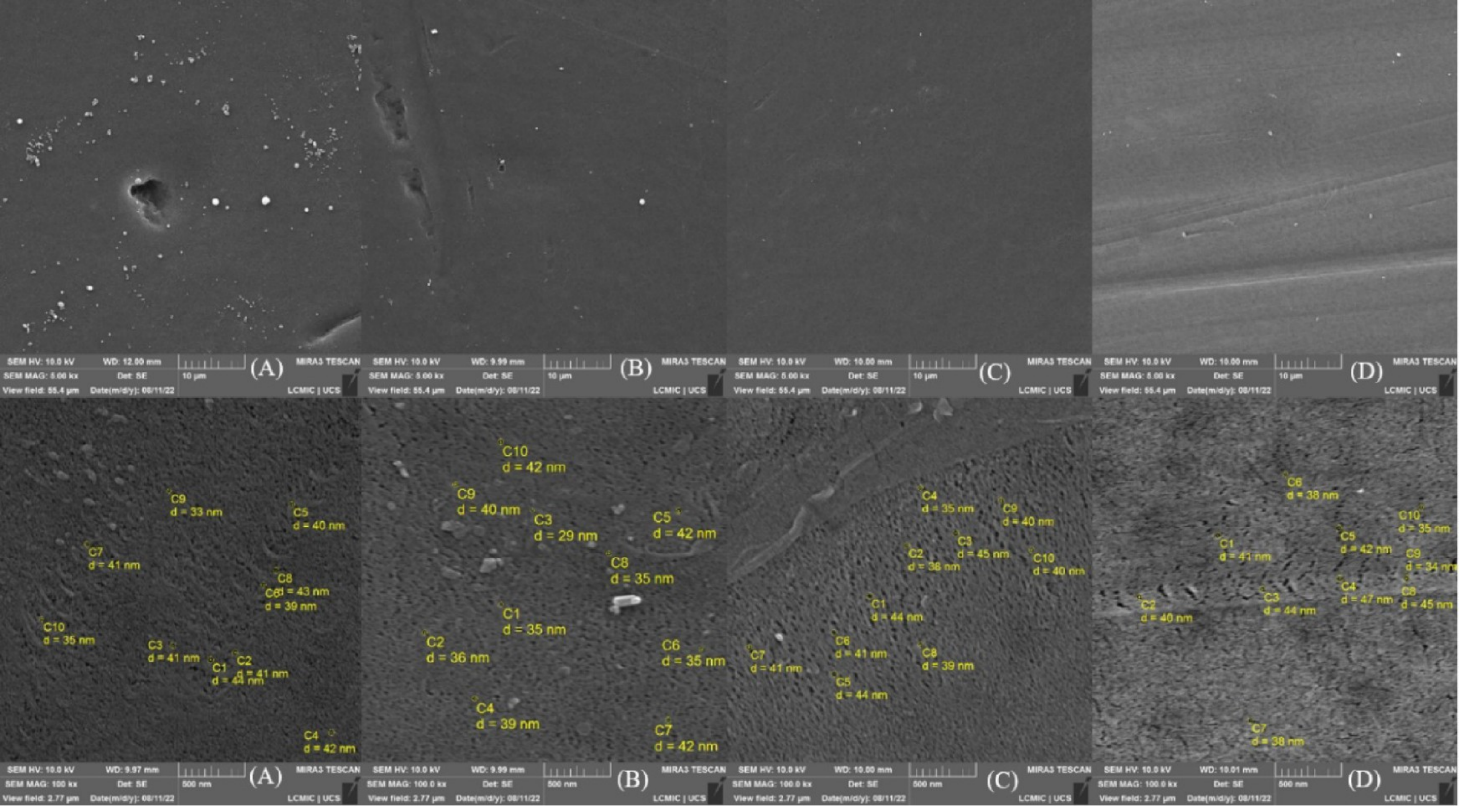
SEM micrographs of the surface of the synthesized membranes, images with magnifications of 5,000× and 100,000×. (A) 0, (B) 0.5 wt % GNP, (C) 1.0 wt % GNP, (D) 1.5 wt % GNP.^16^

**Figure 4:**
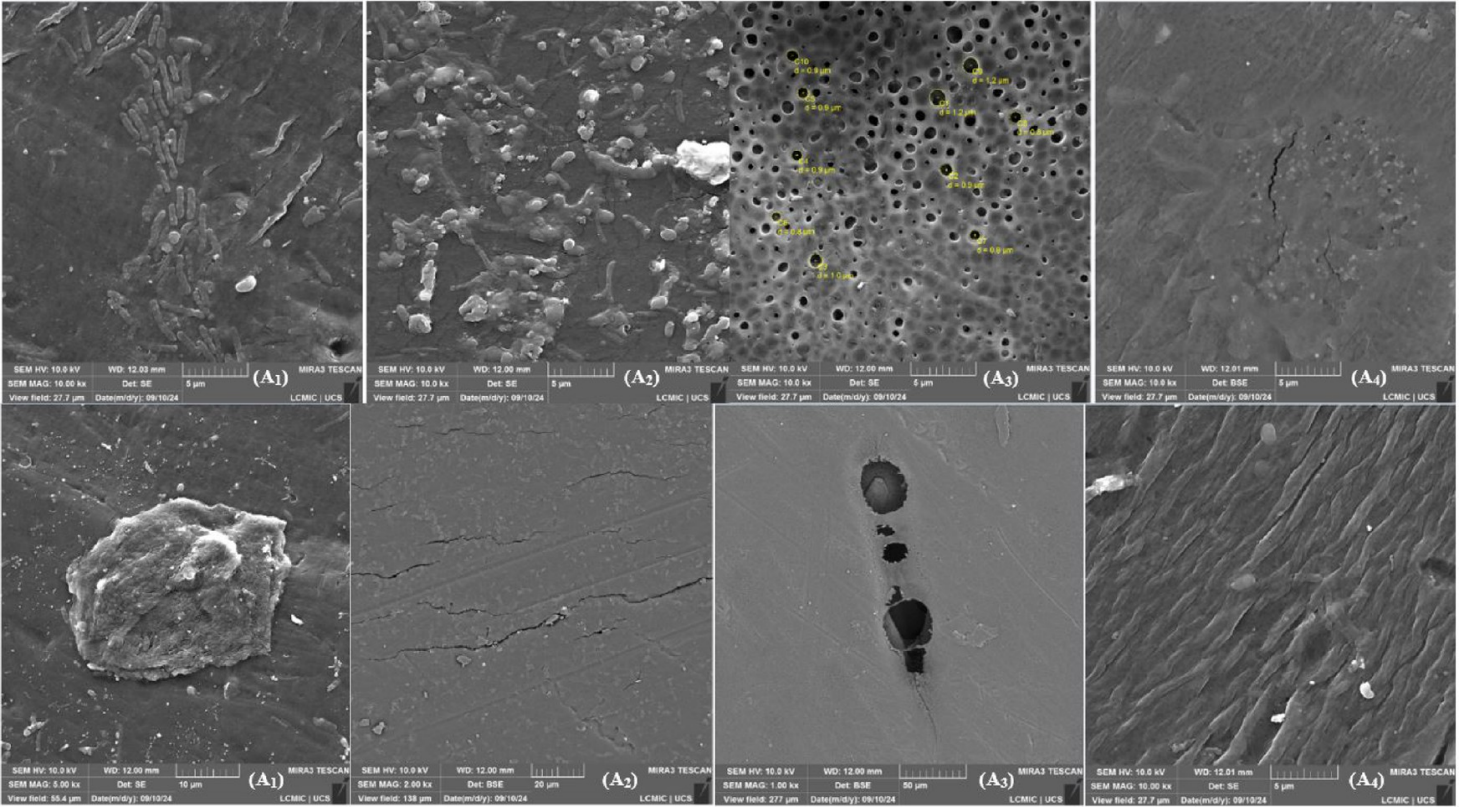
SEM micrographs of the surface of the synthesized membranes, upper images with a magnification of 10,000× and lower images with magnifications of 5,000×, 2,000×, 1,000×, and 10,000×, respectively. (A1) 0, (A2) 0.5 wt % GNP, (A3) 1.0 wt % GNP, (A4) 1.5 wt % GNP.^16^

**Figure 5:**
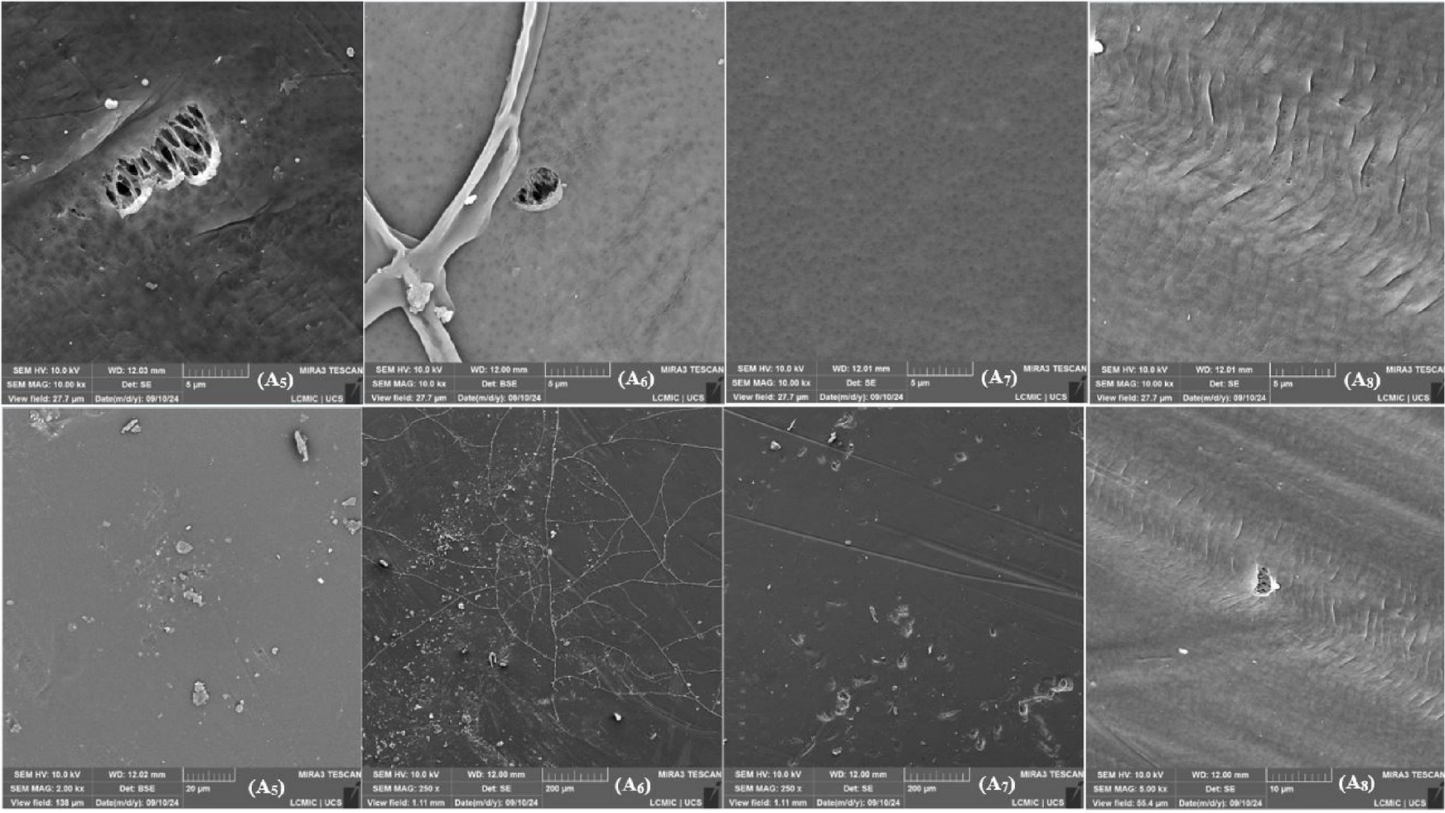
SEM micrographs of the surface of the synthesized membranes, upper images with a magnification of 10,000× and lower images with magnifications of 2,000×, 250×, 250×, and 5,000×, respectively. (A5) 0, (A6) 0.5 wt % GNP, (A7) 1.0 wt % GNP, (A8) 1.5 wt % GNP.

In the 2022 micrographs, pores with an average size of 40 nm were identified, characteristic of an asymmetric structure, as they did not fully traverse the membrane. In contrast, the 2024 micrographs showed a significant change in the surface. In none of the samples and at any magnification was it possible to measure the pores, due to their closure, attributed to the formation of cracks caused by the graphene nanoplatelets (GNP) or the presence of external contaminations.

In micrograph A3, with a magnification of 10,000×, regions containing micropores of approximately 950 nm were identified. These micropores are believed to have originated from cracks and stress regions caused by the GNP flakes. In a 1,000× magnification of the same sample, holes on the membrane surface were visible, reinforcing the influence of the nanoplatelets on the structural modification.

In the micrographs of samples A1 to A4, regions with signs of stress, biological contaminations (possibly bacteria), GNP flakes, and deformations associated with using the membranes in compaction, permeability, and retention tests were observed. In samples A5 to A8, regions with deformations and detachment of the polysulfone matrix, and in sample A6, biological contamination (structures resembling algae or fungal mosaics) was highlighted. These findings indicate that, even when stored in closed conditions, the membranes were exposed to oxygen and moisture, undergoing the effects of structural aging over time.^18,24^

In the cross-sectional micrographs performed in 2022, Figures 6, 7, and 8, NPG flakes and particles attributed to the polysulfone (PSU) matrix were identified. The membrane sheets exhibited a visible separation between layers, characteristic of a less compacted structure. In contrast, the 2024 micrographs, compacted and used samples and pristine pieces, revealed a significant flattening of the membrane layers.

**Figure 6:**
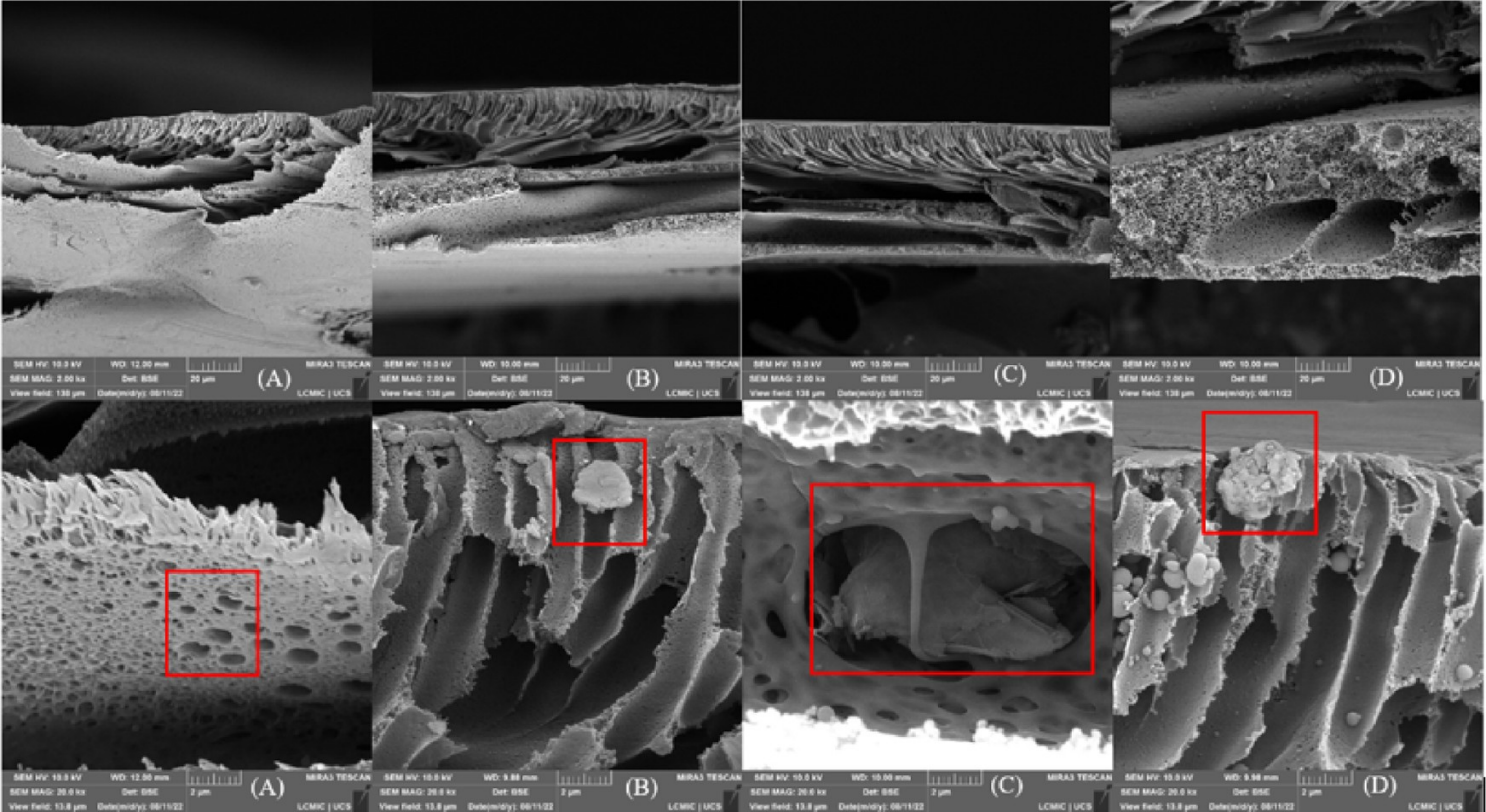
SEM micrographs of the cross-section of the synthesized membranes, upper images with a magnification of 2,000× and lower images with a magnification of 20,000×. (A) 0, (B) 0.5 wt % GNP, (C) 1.0 wt % GNP, (D) 1.5 wt % GNP.^16^

**Figure 7:**
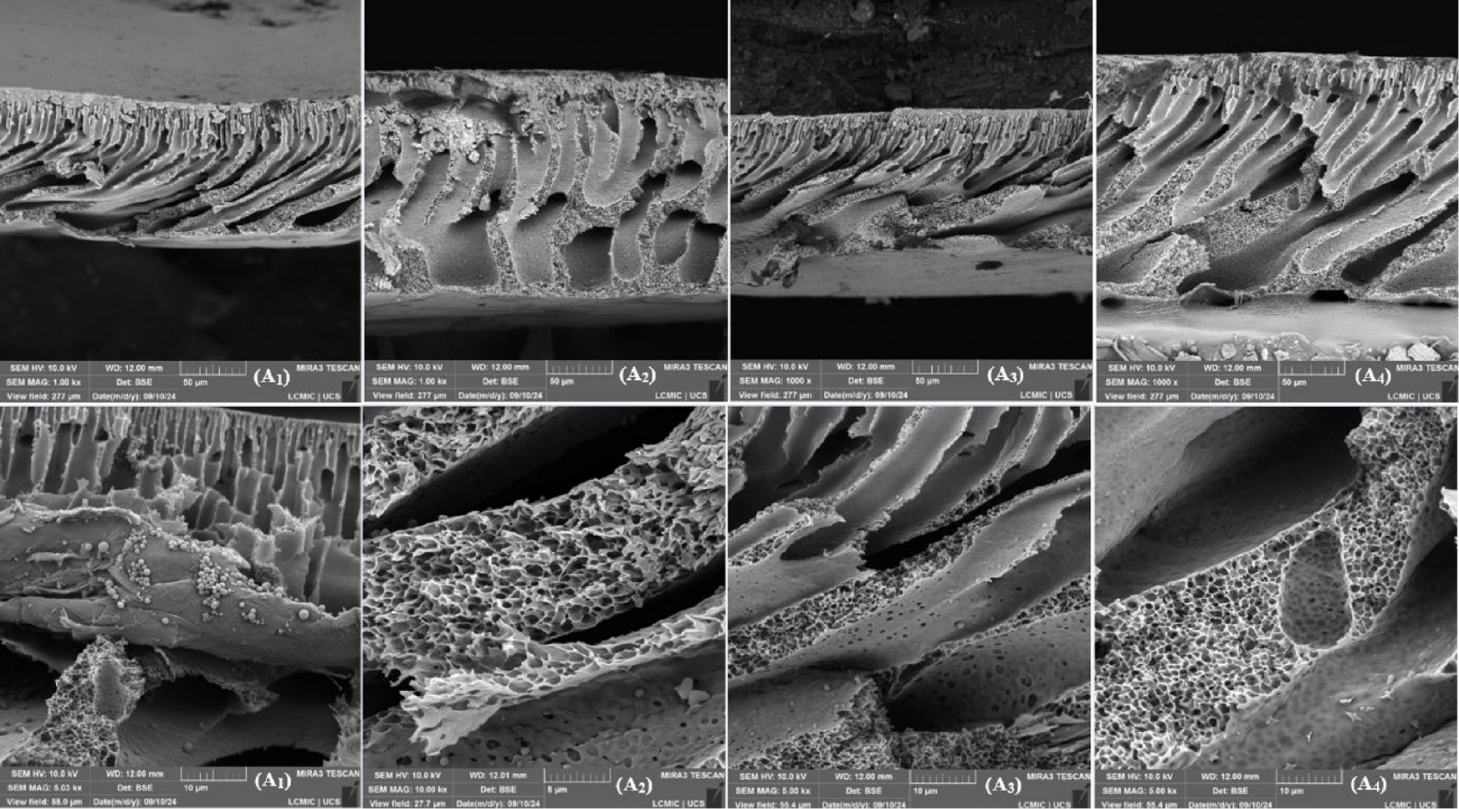
SEM micrographs of the surface of the synthesized membranes, upper images with a magnification of 1,000× and lower images with magnifications of 5,000×, 10,000×, 5,000×, and 5,000×, respectively. (A1) 0, (A2) 0.5 wt % GNP, (A3) 1.0 wt % GNP, (A4) 1.5 wt % GNP.

**Figure 8:**
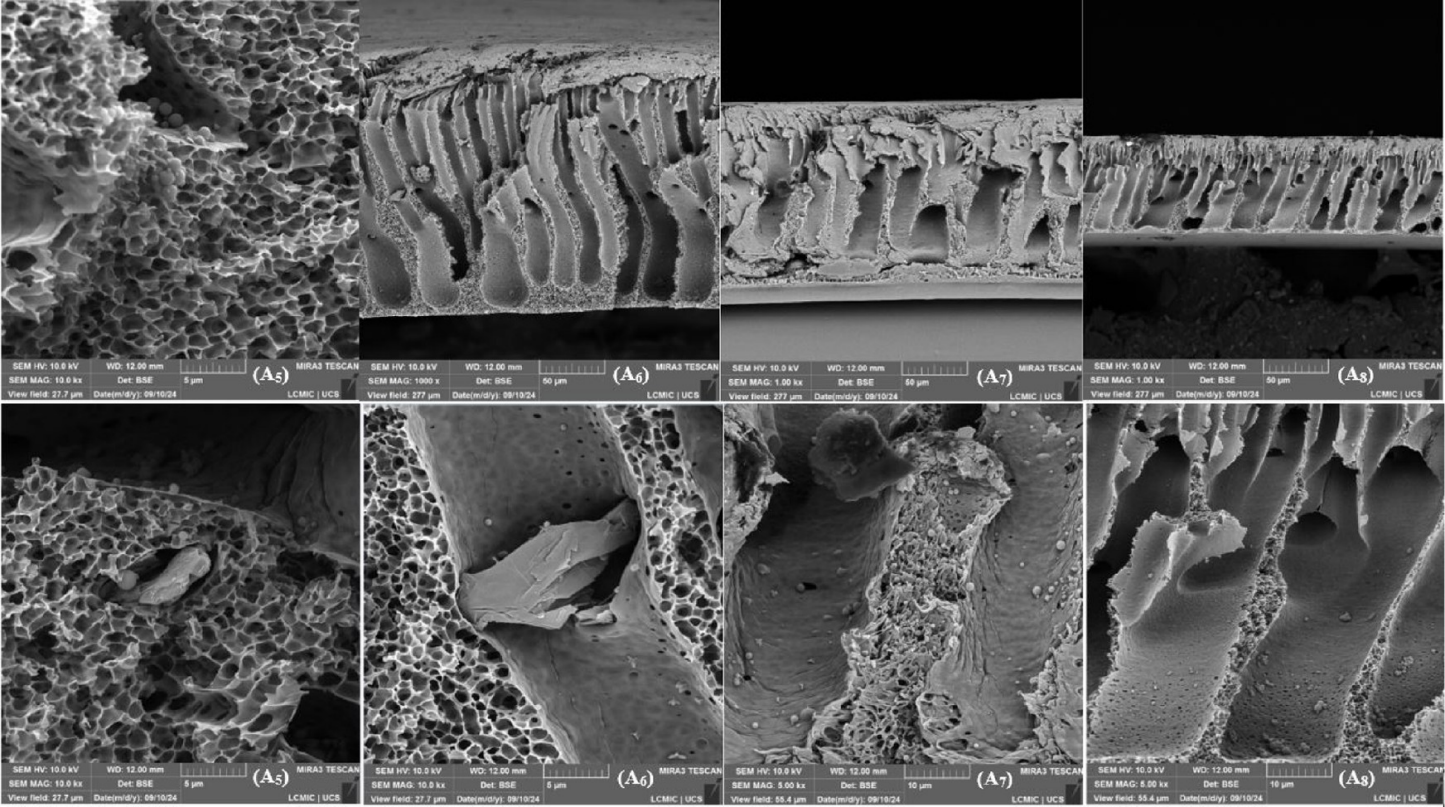
SEM micrographs of the surface of the synthesized membranes, upper images with magnifications of 10,000× and 1,000×, respectively, and lower images with magnifications of 10,000×, 10,000×, 5,000×, and 5,000×, respectively. (A5) 0, (A6) 0.5 wt % GNP, (A7) 1.0 wt % GNP, (A8) 1.5 wt % GNP.

A notable aspect was the increased aggregation of NPG flakes observed in 2024, which aligns with the surface micrographs where the stresses generated by this aggregation were evident. Additionally, all 2024 micrographs, at various magnifications, displayed spherical particles located within the membrane alveoli. Compared with the 2022 micrographs, an increase in the quantity of these particles was noted, both in membranes with and without NPG. This phenomenon was attributed to the degradation of polysulfone, the main structural component of the membrane, caused by aging.

Finally, no significant structural changes were identified with the increase in NPG concentration, indicating that the observed effects are more related to the polymer matrix’s aging and intrinsic degradation than to the additive concentration variation.

The degradation identified by FTIR, characterized by the increase in the intensity of absorption bands, can be corroborated by the results obtained in the 2024 micrographs. Polymer aging can form smaller components, which detach from the polymer matrix and can be visualized through microscopy. These components may be additives or residues, and the migration of some NPG flakes to the membrane surface can be explained by this process.

The polysulfone polymer matrix is also subject to oxidative degradation when exposed to moisture and oxygen, resulting in the fragmentation of the structure and the formation of the spherical particles observed. Another factor that may contribute to the formation of these particles is the residual stress generated during the membrane fabrication process, which remains during storage and causes microcracks, separating particles from the matrix.

Research, such as that of Zheng et al., which investigated the oxidative degradation of polysulfone, also identified spherical particles in the membrane alveoli, indicating degradation promoted by exposure to oxygen and moisture.^25^ Similarly, the review by Ceretti et al. addresses the effects of thermal, oxidative, and mechanical degradation in polymer matrices, confirming the formation of fragments or byproducts that can migrate to the surface of the membranes over time.^26^

### 3.3 Comparison of TG

The TG technique was used to study the thermal degradation of the samples, with TG curves and the maximum point of the curve, obtained by the derivative (DTG), of the eight samples studied in this work compared to the curves from 2022, obtained by Livinalli et al.^16^ Figures 9 and 10 present the 2024 curves of the compacted samples (A1–A4), previously used in 2022 tests, and the curves of the pristine 2024 samples (A5–A8), compared to the TG and DTG curves obtained in 2022, respectively.

**Figure 9:**
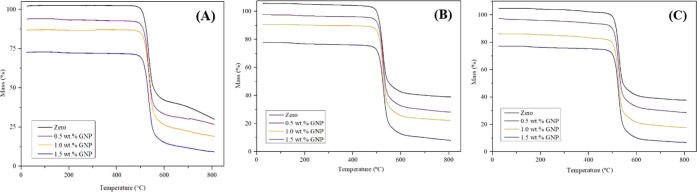
TGA curves of PSU membranes synthesized with different NPG concentrations: (A) TG curves from 2022 – Livinalli et al. (2024), (B) 2024 TG curves of used membranes (A1–A4), (C) 2024 TG curves of unused membranes (A5–A8).^16^

**Figure 10:**
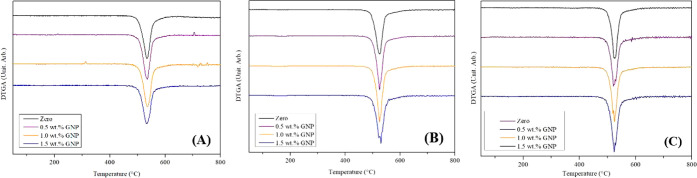
DTG curves of PSU membranes synthesized with different NPG concentrations: (A) DTG curves from 2022 – Livinalli et al. (2024), (B) 2024 DTG curves of used membranes, (C) 2024 DTG curves of unused membranes.^16^

By observing the curves, a similar behavior trend is noticed across all the samples, confirming that the addition of NPG does not result in significant changes in the thermal degradation of the membranes. In 2022, the TG curves showed the onset of mass loss at around 490 °C, corresponding to polysulfone’s degradation point. The maximum degradation point found in the DTG curves was 530 °C, which represents the end point of the mass loss curve.^19,27,28^

Compared with the 2024 curves, both the compacted and unused samples displayed the same behavior, shifting the onset of the mass loss curve to 480 °C, with the curve continuing until 525 °C, the maximum degradation point of the DTG curves. This slight difference can be attributed to differences in the analysis due to the equipment change. However, it can also be associated with a modification in the thermal stability of the membrane, suggesting that the sample might be becoming more susceptible to decomposition due to the aging process or chemical changes related to exposure to the environment, including contact with oxygen, moisture, and light.^29^

### 3.4 BET Analysis

BET tests were conducted on eight membrane samples to validate their classification as ultrafiltration membranes, as determined by Livinalli et al., through the analysis of porosity and surface area.^16^ Additionally, the data from the membranes used in the 2022 compaction and retention tests were compared with the pristine samples. Tables 2 presents the porosity and surface area (BET) data for the compacted membranes (A1 to A4) and the pristine membranes (A5 to A8).

**Table 2:** Results of the membranes’ Average Pore Size and Surface Area Produced in 2022 (A1–A4) and 2024 (A5–A8)

NPG Content	Average pore size (nm)	Surface Area (m^2^ ·g^–1^)
0 (A1)	3.63	322.72
0.5% m/m (A2)	5.49	4.65
1.0% m/m (A3)	3.67	19.30
1.5% m/m (A4)	3.66	10.10
0 (A5)	17.08	5.24
0.5% m/m (A6)	16.43	16.88
1.0% m/m (A7)	17.80	4.69
1.5% m/m (A8)	17.48	6.77

The results indicate that the A3 membrane (1.0 wt % GNP) has a significantly higher surface area than A2 (0.5 wt % GNP) and A4 (1.5 wt % GNP) due to a more developed and interconnected porous structure, maximizing available surface area. In contrast, A2 and A4 likely have larger, less evenly distributed pores, reducing their surface area. Regarding pore size, A2 exhibits larger pores because its lower GNP concentration does not sufficiently regulate phase separation, leading to uncontrolled pore formation. At higher GNP concentrations (A3 and A4), phase separation is more controlled, stabilizing pore size. Once GNP concentration reaches 1.0 wt %, its effect on pore formation saturates, explaining the similar pore sizes in A3 and A4.^30^

The porosity data of both pristine and compacted membranes confirm their classification as ultrafiltration membranes, in accordance with the albumin retention test applied by Livinalli et al. (2024) and the typical characteristics of membranes with pores between 1 and 100 nm.^31^ A comparison between the pristine and compacted membranes shows that the pristine membranes exhibited a porosity ranging from 16.43 to 17.80 nm. In comparison, the compacted membranes had pore sizes between 3.63 and 5.49 nm. This substantial difference can be attributed to the compaction of the structure during the permeability, retention, and compaction tests, in addition to the effects of fouling and scaling, which decrease the porosity due to contamination and impurities.^30,32^

When analyzing the surface area data, there is a low area relative to the pristine membranes, with a variation from 4.69 m^2^·g^–1^ to 16.88 m^2^·g^–1^, presenting fewer larger pores in the overall surface area, exposing less of the membrane’s internal structure. For the used membranes, there is a wide variation from 4.65 m^2^·g^–1^ to 322.72 m^2^·g^–1^, where the compaction of the pores may redistribute the polymer matrix structure by decreasing the pore size, thus increasing the available surface area. In this case, the surface area is larger for the membranes without NPG due to the nanoparticles agglomerating, which reduces the exposed surface. Therefore, NPG influences the stability of the membrane’s porous structure, with less redistribution of pores after compaction or use of the samples.^30^ In the research of Shen et al., membranes synthesized with reduced graphene oxide were studied, and the membranes had a pore size of less than 10 nm. The study also helped in understanding how nanoparticle agglomeration reduces the available surface area.^30^

### 3.5 Comparison of Permeability

Permeability tests were carried out on all eight samples (A1 to A8), varying the pressure from 1 to 6 bar for the compacted membranes. This allowed the determination of permeability values for membranes A1, A2, and A3. During the testing process, several limitations were identified that impacted the reliability of some of the obtained data, membranes A5 to A8 did not withstand the compaction tests, and membrane A4 did not survive the permeability tests, showing ruptures. Given these limitations, it is important to clarify that the obtained data cannot be reliably replicated, and the results should be interpreted cautiously. We recommend that future studies explore alternative testing methods to overcome these limitations and validate the observed results.

Tables 3 displays the permeability (*L*_p_) values for membranes A1 to A4 obtained by Livinalli et al. (2024), compared with those obtained in 2024.

**Table 3:** Permeability Values (*L*_p_) of Membranes Produced with the Addition of 0, 0.5 wt %, 1.0 wt %, and 1.5 wt % of Graphene Nanoplatelets^a^

NPG Content	Permeability 2022 (L·m^–2^·h^–1^·bar^–1^)	Permeability 2024 (L·m^–2^·h^–1^·bar^–1^)
0	6.78 ± 1.82	0.010
0.5% m/m	2.44 ± 0.65	0.197
1.0% m/m	0.32 ± 0.04	0.013
1.5% m/m	0.10 ± 0.04	-

a Source: adapted from Livinalli et al. (2024).

The data from 2022 were obtained in triplicate, while in 2024, the membranes did not survive the tests, making it difficult to obtain reliable data. The rupture of the membranes can be attributed to the fragility induced by the agglomerated NPG flakes observed in the micrographs of the membranes, particularly at high NPG concentrations (1.5 wt %). This effect compromised the structural uniformity, corroborating the surface area results obtained in the BET analysis, indicating greater membrane fragility when subjected to pressure.

The pristine membranes, when exposed to oxygen and moisture, may have undergone degradation of the essential chemical bonds, weakening the mechanical structure of the membrane. Time may have also induced plasticity in the polymer matrix, making it more rigid and fragile. Additionally, the larger pores identified in the BET analysis (16 nm) in the pristine membranes may be responsible for the lower resistance to compaction due to the reduced mechanical support.

By comparing the permeability data from 2022 with those from 2024, the degradation of the polymer matrix over time is evident, as shown by the MEV, FTIR, TGA, and BET analyses. The permeability of the control (zero NPG) decreased drastically from 6.78 L·m^–2^·h^–1^·bar^–1^ to 0.01 L·m^–2^·h^–1^·bar^–1^, reflecting severe degradation, along with fouling and compaction effects. The membranes with 0.5% m/m NPG exhibited the smallest reduction in permeability, from 2.44 L·m^–2^·h^–1^·bar^–1^ to 0.1971 L·m^–2^ ·h^–1^·bar^–1^, indicating greater structural stability over time; however, the membranes with 1.0 and 1.5 wt % NPG showed greater sensitivity to degradation, suggesting that time affected their permeability more significantly.^32^

## 4 Conclusion

The study demonstrated that time negatively affects the structure and performance of PSU membranes with NPG. The compacted membranes exhibited greater structural stability due to pore redistribution but suffered a significant reduction in permeability. The pristine membranes were more susceptible to rupture, showing mechanical and chemical fragility. Optimizing storage and exploring strategies to mitigate degradation is essential to improving membrane durability and performance. Future studies may include chemical modifications to the polymer matrix and long-term evaluations under controlled conditions. With graphene, a lifespan of more than 10 years was expected for the membranes. However, based on the effects of aging demonstrated in this study, it is necessary to investigate alternative storage methods to ensure their long-term stability and performance.
